# Structural and functional characterization of salmon STAT1, STAT2 and IRF9 homologs sheds light on interferon signaling in teleosts

**DOI:** 10.1016/j.fob.2014.09.007

**Published:** 2014-10-07

**Authors:** Mehrdad Sobhkhez, Astrid Skjesol, Ernst Thomassen, Linn Greiner Tollersrud, Dimitar B. Iliev, Baojian Sun, Børre Robertsen, Jorunn B. Jørgensen

**Affiliations:** The Norwegian College of Fishery Science, UiT The Arctic University of Norway, N-9037 Tromsø, Norway

**Keywords:** GAF, gamma interferon activation factor, IAD, IRF-association domain, IFN, type I interferon, IRF, interferon regulatory factor, ISGF3, IFN-stimulated gene factor 3, ISRE, IFN-stimulated response element, NES, nuclear export signal, NLS, nuclear localization signal, TAD, transcriptional activation domain, Atlantic salmon, STAT2, IRF9, IFN, Fish

## Abstract

Mammalian IRF9 and STAT2, together with STAT1, form the ISGF3 transcription factor complex, which is critical for type I interferon (IFN)-induced signaling, while IFNγ stimulation is mediated by homodimeric STAT1 protein. Teleost fish are known to possess most JAK and STAT family members, however, description of their functional activity in lower vertebrates is still scarce. In the present study we have identified two different STAT2 homologs and one IRF9 homolog from Atlantic salmon (*Salmo salar*). Both proteins have domain-like structures with functional motifs that are similar to higher vertebrates, suggesting that they are orthologs to mammalian STAT2 and IRF9. The two identified salmon STAT2s, named STAT2a and STAT2b, showed high sequence identity but were divergent in their transactivation domain (TAD). Like STAT1, ectopically expressed STAT2a and b were shown to be tyrosine phosphorylated by type I IFNs and, interestingly, also by IFNγ. Microscopy analyses demonstrated that STAT2 co-localized with STAT1a in the cytoplasm of unstimulated cells, while IFNa1 and IFNγ stimulation seemed to favor their nuclear localization. Overexpression of STAT2a or STAT2b together with STAT1a activated a GAS-containing reporter gene construct in IFNγ-stimulated cells. The highest induction of GAS promoter activation was found in IFNγ-stimulated cells transfected with IRF9 alone. Taken together, these data suggest that salmon STAT2 and IRF9 may have a role in IFNγ-induced signaling and promote the expression of GAS-driven genes in bony fish. Since mammalian STAT2 is primarily an ISGF3 component and not involved in IFNγ signaling, our finding features a novel role for STAT2 in fish.

## Highlights

• The transcription factors STAT2 and IRF9 from Atlantic salmon have been identified.
• STAT2 co-expressed with STAT1 translocates to the nucleus upon IFN stimulation.
• STAT2 are tyrosine phosphorylated upon type I IFN and IFNγ stimulation.
• STAT2 and STAT1, as well as IRF9, activates GAS elements upon IFNγ stimulation.
• Our results suggest novel roles of salmon STAT2 and IRF9 in IFNγ-mediated signaling.

## 1 Introduction

Known from many studies in mammals, Signal Transducer and Activator 1 and 2 (STAT1 and STAT2) are key mediators of type I interferon (IFN) signaling and they are essential components of the cellular antiviral response and adaptive immunity. Both are latent transcription factors that usually are situated in the cytoplasm, and travel to the nucleus in response to IFN stimulation. STAT1 and STAT2 are activated by Janus Kinase (JAK) molecules that are auto and/or transactivated after multimerization of IFN receptors as reviewed in [1,2]. First, JAK activity leads to phosphorylation of tyrosine residue(s) on the receptors intracellular domains [3–5], then the STAT molecules dock on these phosphorylated sites [6] and they become activated after being tyrosine phosphorylated by the JAKs [7,8]. Phosphorylated STAT1 and STAT2 heterodimer and together with the interferon regulatory 9 (IRF9), translocate to the nucleus and form a stable complex named IFN-stimulated gene factor 3 (ISGF3) [9] on the IFN-stimulated response element (ISRE) in the promoters of responsive genes [10]. STAT1 also has a prominent role in the IFNγ signaling, where engagement with the IFNγ receptor leads to STAT1 phosphorylation and dimerization. The STAT1 homodimer, also called gamma interferon activation factor (GAF), translocates to the nucleus and binds to an IFNγ stimulated gene response element (GAS) and activates transcription of responsive genes [11,12]. Targeted gene disruptions of STAT1, STAT2, or IRF9 clearly demonstrate that animals lacking one of the factors are highly susceptible to diverse viruses and to some intracellular bacterial pathogens [13–18].

**Fig. 1:**
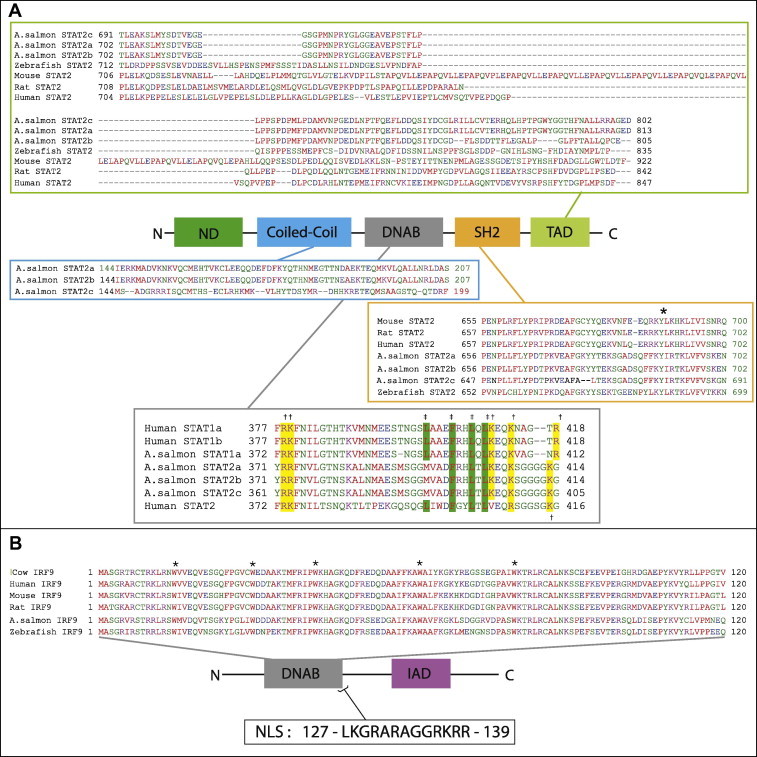
Salmon STAT2 proteins harbor conserved domains and sequences. (A) The NCBI conserved domains database (CDD) and ClustalW alignment showed presence of conserved domains; N-terminal domain (ND), Coiled-coil, DNA binding domain (DBD), SH2 homology domain (SH2) and transcription activation domain (TAD). ClustalW alignment revealed high aa identity between the three identified salmon STAT2 sequences (STAT2a KJ155789, STAT2b KJ155790 and STAT2c FJ173070) and the highest divergence between STAT2c and STAT2a/STAT2b was observed in their Coiled-coil domain (aa 144–199/207). A conserved tyrosine residue (Y) which is a target of phosphorylation by the JAKs is marked with *. DNAB domain of STAT possess conserved aa essential for nuclear localization (marked yellow with †) and nuclear export (marked green with ‡). (B) DNAB domain of Atlantic salmon IRF9 has high sequence identity with IRF9 DNAB domains from other species. ClustalW alignment using the NCBI CDD of the deduced aa sequences of IRF9 DNAB domain from Atlantic salmon (NP_001167190), human (NP_006075), cow (AAI02048), mouse (AAH05435), rat (NP_001012041 XP_224190) and zebrafish (AAH81591). Asterisk (*): indicate conserved tryptophan (W) residues. (For interpretation of the references to colour in this figure legend, the reader is referred to the web version of this article.)

STATs have a conserved architecture (Fig. 1) and consist of an N-terminal domain that makes it possible for dimerized STAT to polymerize and bind cooperatively to DNA [19,20], a coiled-coil domain that facilitates interactions to other proteins, followed by a DNA-binding domain (DNAB), and a linker domain that is important for transcription [21,22]. The C-terminal part has a Src-homology 2 (SH2) domain that interacts with receptors [8]. The latter includes a tyrosine phosphorylation site, and the SH2 domain is the domain essential for dimerization. Finally the STATs have a transcriptional activation domain (TAD) which mediates their transcriptional activity [23]. IRF9 is a member of interferon regulatory factor (IRF) family and has an established role in type I IFN responses. This molecule too has a conserved structure which is comprised of a well-defined DNAB in addition to an IRF-association domain (IAD) [24]. The DNAB domain is highly conserved across different species and includes 5 conserved tryptophan residues, while the IAD is much more diverse and works as regulatory domain known to associate with distinct transcription factors [25].

A great deal of research has been directed towards deciphering IFN-induced signaling in bony fish reviewed in [26–32] and in earlier reports our group has identified Atlantic salmon (*Salmo salar*) homologs to STAT1 and Tyk2 and provided data demonstrating functional activity of these molecules [33,34]. The present work was initiated to extend these studies aimed at extending the knowledge about the JAK–STAT signaling in teleost fish.

Here we report the cloning and identification of two STAT2 (STAT2a, STAT2b) homologs and one IRF9 homolog from Atlantic salmon. We show that the expression of all three proteins is modulated upon stimulation of salmon cell lines with type I and type II IFNs. The two STAT2s do interact with STAT1a [33], with ssTYK2-1 [34] and with IRF9 and they are tyrosine-phosphorylated upon IFN-treatment. In earlier studies we have shown that STAT1a resides mainly in the cytoplasm of unstimulated cells, while IFNγ stimulation results in a nuclear accumulation [33]. Here we studied the cellular localization of overexpressed STAT2a and b alone or in combination with STAT1a and IRF9 in untreated and IFNa1 and IFNγ stimulated TO cells. While IRF9 was targeted to the nucleus in absence of IFN stimulation, STAT2a and STAT2b remained in the cytoplasm of unstimulated cells. However, when the two STAT2s were co-expressed with IRF9, these complexes, independent of IFN stimulation, were found both in the nucleus and cytoplasm. Both STAT2a and STAT2b co-localized with STAT1a in the cytoplasm of unstimulated cells, while upon IFN stimulation the STAT1a and STAT2 proteins translocated to the nucleus. Finally, we present evidence that the combination of STAT2 and STAT1, as well as IRF9, activates GAS elements upon IFNγ stimulation, thus suggesting involvement of these factors in mediating type II IFN responses in teleost fish.

## 2 Results

### 2.1 Comparison of salmon STAT2 amino acid sequence and phylogenetic analysis

**Fig. 2:**
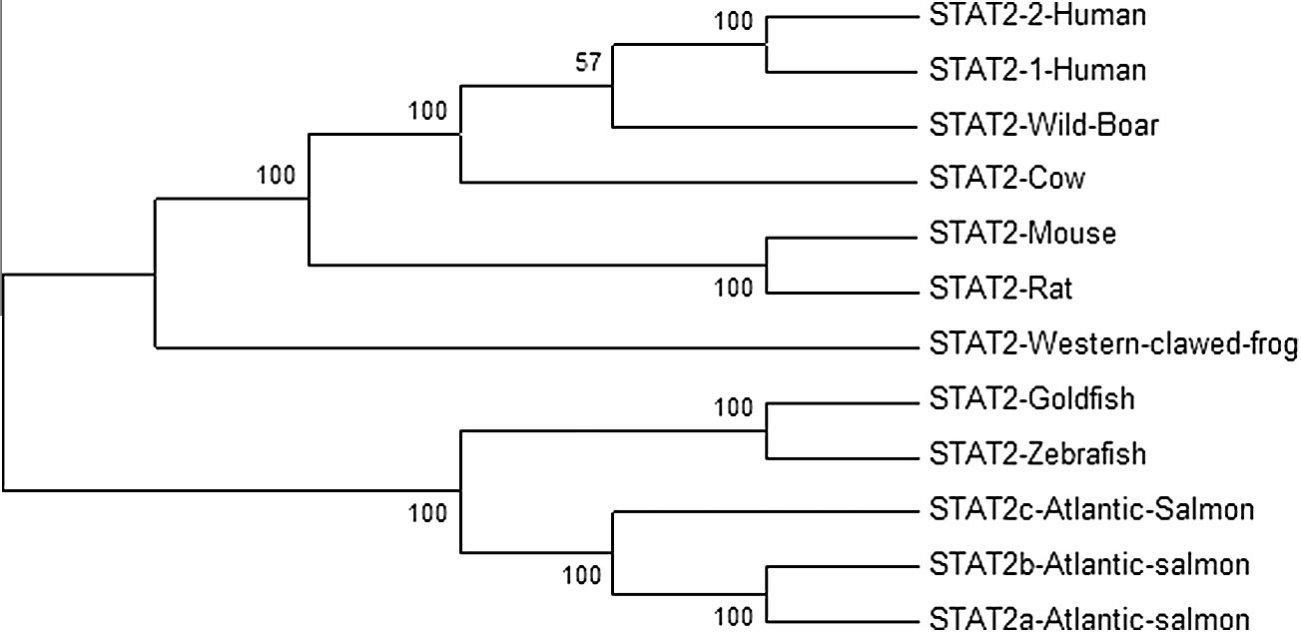
A phylogenetic tree of STAT2 protein sequences in Atlantic salmon and selected vertebrate species. The phylogenetic tree was constructed by the neighbor-joining method in the MEGA 5 program and the bootstrap values were calculated with 500 replicates. STAT2 from following species are included: Cow, goldfish, zebrafish, human, mouse, rat, wild boar, western clawed frog, and the three STAT2 isoforms of Atlantic salmon. GenBank accession numbers of the sequences are listed in Table 1.

Sequencing and analysis of the cloned PCR products obtained by the STAT2 primers (Table 1) revealed two fragments with open readings frames of 2445 bp (STAT2are, GenBank accession no. KJ155789) and 2418 bp (STAT2b, GenBank accession no. KJ155790) encoding for putative polypeptides of 813 amino acid (aa) and 805 aa, respectively. Alignment of aa sequences (ClustalW2) showed that STAT2a and STAT2b had a 96% aa identity to each other and their best match (92% and 88% aa identity, respectively) was to an earlier reported Atlantic salmon STAT2 variant (FJ173070) [35]. We propose that the previously published salmon STAT2 (FJ173070) to be referred to as salmon STAT2c. Alignment of the STAT2a and STAT2b with STAT2-sequences from other species showed 57% and 58% aa identities with zebrafish STAT2, while their identities to different mammalian STAT2s were close to 40% (Table 3). In the phylogenetic tree presented in Fig. 2, STAT2a and STAT2b are placed in the same clad and grouped with other fish species.

### 2.2 Domain organization and characterization of salmon STAT2

The analysis of conserved domains using NCBI CDD [37] showed that STAT2a and STAT2b displayed conserved domains found in STAT2 molecules from other species including a N-terminus, a coiled-coil domain, a DNAB domain, a SH2 domain and a C-terminal TAD-domain (Fig. 1A). The TAD-domain was highly conserved between salmon STAT2a and STAT2c, while both molecules displayed higher divergence to STAT2b (Fig. 1A). In the SH2 domain a conserved tyrosine phosphorylation site was identified in both STAT2a and STAT2b (Y690) and also in STAT2c. This site corresponds to a tyrosine found in exactly the same position in human STAT2-1 [44]. The SH2 domain was followed by linker domain and then a DNAB domain, which is responsible for interaction with DNA. This domain showed high aa sequence similarity between all three salmon STAT2 sequences especially in one segment (aa 371–414 in STAT2a/b) which was 100% conserved between STAT2a, b and c and also had very high aa identity with human STAT2 (Fig. 1A). Interestingly this is the domain where the latent nuclear localization signal (NLS) for human STAT1 and STAT2 are reported to reside [45]. As reported for human STAT1, STAT molecules do not have a classical NLS sequence but they do possess a segment that is rich in basic amino acids located in or close to their DNAB domain. In human STAT1 this segment includes the residues Arg378, Lys379, Lys410, Lys413, and Arg418. These residues are conserved between human and salmon STATs, where salmon STAT1a and the three STAT2s possess Arg372, Lys373, Lys404, Lys407, while Arg412 (corresponding to Arg418 in human STAT1) was only present in salmon STAT1a and not found in human and salmon STAT2. Furthermore, a lysine placed in a position shown to be essential for human STAT2 nuclear localization [45] was conserved between salmon (position 412) and human STAT2.

In the same segment of the STAT DNAB domain (aa 392–413 in human STAT1) several hydrophobic residues are located. This segment is almost identical between human STAT1 and salmon STAT1a, with the exception of Thr396 in human STAT1 which is lacking in salmon (Fig. 1A). Hydrophobic residues in this segment of human STAT1 include Leu400, Phe404, Leu407 and Leu409, and replacement of any of these residues with non-hydrophobic residues is shown to eliminate human STAT1 nuclear export activity, which identifies this sequence as a nuclear export signal (NES) [46]. ClustalW alignment showed that these residues correspond to salmon STAT1a Leu394, Phe398, Leu401 and Leu403. In STAT2a and STAT2b, the Phe398, Leu401 and Leu403 residues are present at the same positions as in STAT1a (Fig. 2B). The coiled-coil domain displayed high similarity between STAT2a and b, whereas the highest divergence between these two and salmon STAT2c (GenBank accession no. FJ173070) was found here (Fig. 1A). And finally, N-terminal domain that was 100% identical between all three salmon STAT2 sequences (data not shown).

### 2.3 Cloning and characterization of salmon IRF9

A PCR-product of full-length salmon IRF9 cDNA was amplified using primers (Table 1) based on salmon IRF9 sequence (GenBank accession no. NM_001173719.1) [36]. The analysis of the sequence revealed a 1293 bp ORF encoding a 430 aa polypeptide. The study of functional domains using NCBI CDD [37] revealed structures characteristic for IRF9 including a well-defined DNAB as well as an IAD [24] (Fig. 1B). Salmon IRF9 showed 30% to 35% aa identity with different mammalian IRF9s and 55% aa identity with zebrafish IRF9. The most divergent part of the molecule when compared to other species was the IAD-domain where the aa identity to zebrafish was less than 35% and to the mammalian species included in the analysis was less than 19%. The DNAB domain (aa 1–120) was highly similar to the zebrafish DNAB (82% aa identity), while the identity to mammalian DNAB was 65%. Within the salmon DNAB five conserved tryptophan residues (W15, 30, 42, 62 and 80) were identified in the same positions with those found in zebrafish and mammalian IRF9 (Fig. 1B). Atlantic salmon IRF9 sequence included a strong NLS (127 – LKGRARAGGRKRR – 139) detected next to the DNAB domain (Fig. 1B).

### 2.4 Expression of STAT2a, STAT2b and IRF9 in organs of healthy Atlantic salmon

**Fig. 3:**
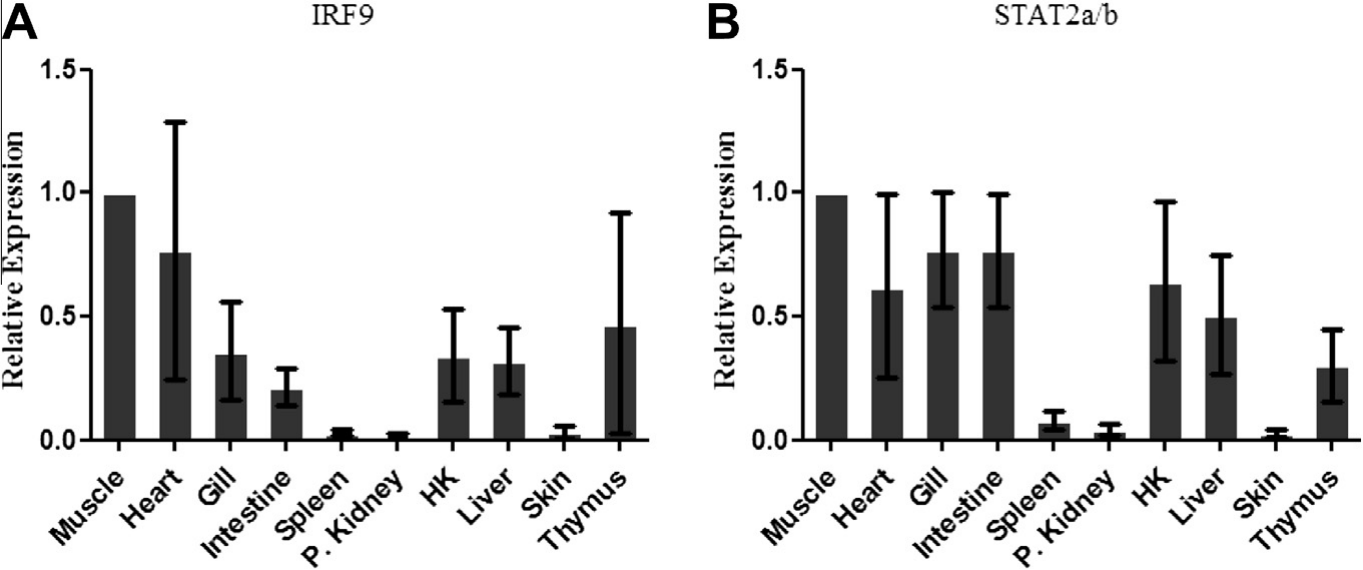
Relative gene expression of STAT2a/b and IRF9 in tissues from healthy Atlantic salmon The relative expression of STAT2a/b (A) and IRF9 (B) in the tissues heart, gill, intestine, spleen, posterior kidney (P. Kidney), head kidney (H. K), liver, skin, thymus and muscle were measured by qPCR and presented relative to EFlαβ by the formula (ctEFlαβ)/(ct of STAT2 or IRF9) Samples from 6 fish were analyzed and error bars represents the mean ±SD.

Tissues taken from healthy Atlantic salmon (heart, gill, intestine, spleen, posterior kidney (P. Kidney), head kidney (HK), liver, skin, thymus and muscle) were analyzed by qPCR using primer pairs detecting both STAT2a/b isoforms and IRF9 (Table 1). Results showed that STAT2a/b and IRF9 were expressed in all tissues tested. STAT2a/b revealed the highest relative expression in heart, gill, intestine, HK, muscle and liver, while significantly lower expression (*p* > 0.05) was found in spleen, P. Kidney and skin (Fig. 3A).

The expression of IRF9 was significantly higher in heart and muscle compared to the other tissues that were included in this study. The lowest expression of IRF9 was found in spleen, P. Kidney, and skin (Fig. 3B), and the expression in these 3 organs were significantly lower compared to the other tissues examined.

### 2.5 STAT1a, STAT2 a and STAT2b are tyrosine phosphorylated upon IFN stimulation

**Fig. 4:**
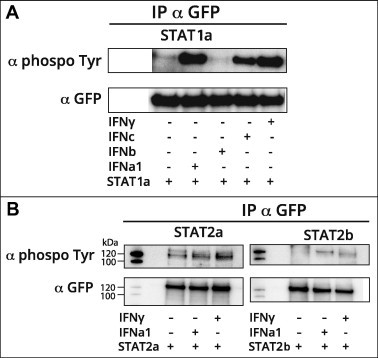
Atlantic salmon STAT1a, STAT2a and STAT2b are tyrosine phosphorylated upon IFN stimulation. (A) CHSE-214 cells were transfected with plasmids expressing GFP-tagged STAT1a and either were left unstimulated or were stimulated with 200 U/ml IFNa1, b and c and 500 ng/ml IFNγ for 30 min before harvest. (B) CHSE-214 cells were transfected with GFP-tagged STAT2a and STAT2b. The cells were either left unstimulated or they were stimulated with 200 U/ml IFNa1, and 500 ng/ml IFNγ for 30 min before harvest. IP was performed with anti-GFP antibody and tyrosine-phosphorylated proteins were detected by Western blot using anti-phosphotyrosine antibody. The membranes were stripped and re-probed with anti-GFP antibody to detect the total amount of immunoprecipitated proteins. These experiments were repeated 3 times with the same result (results not shown).

Tyrosine phosphorylation is a key step in STAT-mediated IFN signal transduction and both salmon STAT1a [33] and the two salmon STAT2 isoforms identified in this study possess phosphorylation sites that are conserved between salmon and human. The conventional view is that type I IFN signals through STAT1/STAT2 heterodimers while IFNγ signals through STAT1 homodimers. In Atlantic salmon 4 different type I IFN subtypes are identified, IFNa, b, c and d, and recent studies have revealed distinct roles for the different subtypes [41]. In an earlier study we have shown that endogenous STAT1a is phosphorylated upon stimulation with IFNa1 and IFNγ [33]. Here, we extended the studies and tested tyrosine phosphorylation of overexpressed STAT1a, when stimulated with recombinant IFNa1, IFNb, IFNc and IFNγ in CHSE-214 cells. IFNa1 and IFNγ were also tested for their ability to phosphorylate both STAT2a and STAT2b. The GFP-tagged STAT expressing plasmids were transfected in CHSE-214 and transfectants were stimulated with IFNs. The transfectants were harvested after 30 min of stimulation with the IFNs and anti-GFP antibody was used to immune precipitate the various overexpressed GFP-tagged STAT proteins. The results were visualized by Western blotting. As shown in Fig. 4A STAT1a is phosphorylated when stimulated with IFNa1, IFNc and IFNγ, while no phosphorylation was detected for cells stimulated with IFNb. Phosphorylated STAT2a and b were detected after 30 min stimulation by IFNa1 and also, very interestingly, by IFNγ (Fig. 4B). The phosphorylation was not as strong as observed for STAT1a, but still higher when compared to the unstimulated control.

### 2.6 Salmon STAT1a is phosphorylated on a single tyrosine (Tyr 695) residue upon activation by IFNs

**Fig. 5:**
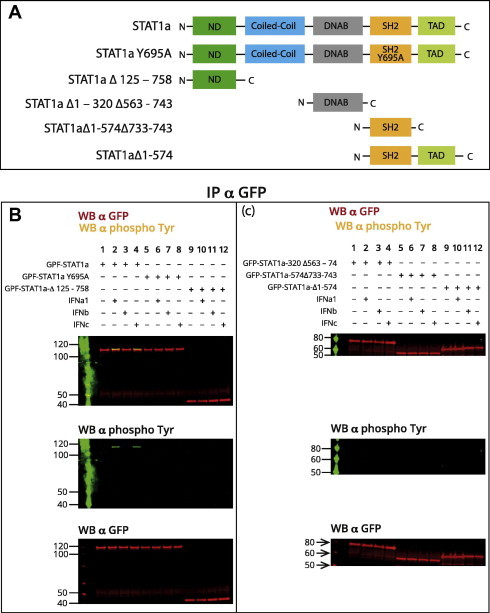
Salmon STAT1a is phosphorylated on a single site (Tyr 695) in the SH2-domain in type I IFN-stimulated cells and this phosphorylation requires expression of full-length STAT1a. (A) Constructs used for immunoprecipitations: full-length STAT1a, STAT1a Y695A and 4 different deletion mutants. (B) CHSE-214 cells were transfected with plasmids encoding different GFP-tagged STAT1a, STAT1a deletion mutants or point mutated STAT1a Y695A. The cells were harvested 72 h post transfection, and 30 min prior to harvest they were stimulated with 200 U/ml of IFNa1, b or c. Cell extracts were immunoprecipitated with anti-GFP antibody and tyrosine-phosphorylation was detected by immunoblotting using α-phospho-tyrosine antibody. The membrane was stripped and re-probed with α-GFP to detect the total amount of immunoprecipitated proteins.

Next, we wanted to test the functional importance of the tyrosine 695 in salmon STAT1a [33] and we made a construct where this tyrosine was replaced with alanine (STAT1a Y695A) and tested it together with wild-type STAT1a for the abilities of these two constructs to be phosphorylated upon IFN-treatments. Additionally, different STAT1a deletion constructs (Fig. 5A) were made and also tested in a similar manner.

**Fig. 6:**
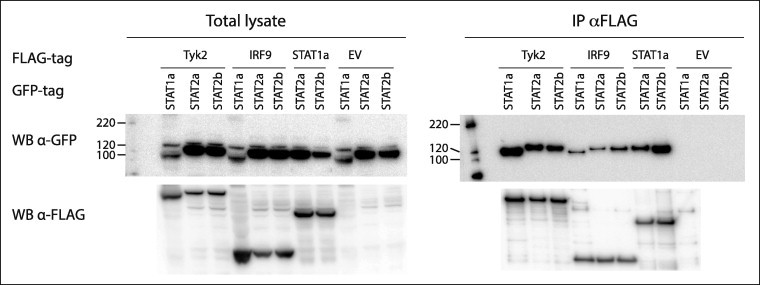
STAT1a interacts with Tyk2 and IRF9, and STAT2a and STAT2b interact with Tyk2, IRF9 and STAT1a. HEK 293 cells co-transfected with plasmids expressing TYK2, IRF9 or STAT1a with FLAG-tag in combination with plasmids expressing either STAT2a or STAT2b with GFP-tag. The study also included plasmids expressing FLAG-tagged Tyk2 or IRF9 in combination with STAT1a with GFP-tag. The IP was performed with anti-FLAG M2 affinity gel and interacting partners were detected by Western blot using antibodies against GFP, membrane was stripped and treated with anti-FLAG antibody. An empty pDEST-3xFlag vector was included as control. All the proteins were shown to be expressed in total lysate.

The results presented in Fig. 6B show that while wild-type STAT1a was tyrosine phosphorylated upon IFNa1 and IFNc treatment, the STAT1aY695A mutant showed no phosphorylation in response to the same stimuli. As presented in Fig. 6B, the phosphorylated form of STAT1 (green band) migrated slightly slower than the non-phosphorylated form (red band). These results suggest STAT1a is phosphorylated on a single site (tyrosine 695). No tyrosine phosphorylation was detected on constructs expressing the SH2 domain alone or the SH2 in combination with TAD domain (Fig. 5C), which was surprising since tyrosine 695 is located in the SH2 domain. The same experiment as presented in Fig. 6 was also set up with IFNγ stimulation and gave similar results (results not shown).

### 2.7 Salmon STAT2a and STAT2b bind to STAT1a, IRF9 and Tyk2-1

In mammals STAT1 and STAT2 interactions with Tyk2 is necessary for their tyrosine phosphorylation, which in turn is required for their heterodimerization. IRF9 also interacts with the STAT1–STAT2 heterodimer and the three factors together constitute the ISGF3 [44]. Co-IP studies using tagged variants of ssTyk2-1, STAT1a, STAT2a, STAT2b, and IRF9, revealed interaction patterns similar to their mammalian counterparts [9]. As shown in Fig. 6 STAT1a interacted with STAT2a, STAT2b, IRF9 and ssTyk2-1, also STAT2a and STAT2b did interact with IRF9 and ssTyk2-1. In these studies a 3XFLAG empty vector was included as a control and no interactions were found between this vector control and the constructs tested. This study was repeated with different tag and IP both ways, with the same results (result not shown).

### 2.8 STAT2a and STAT2b reside in the cytoplasm, whereas IRF9 localize to nucleus of TO cells

**Fig. 7:**
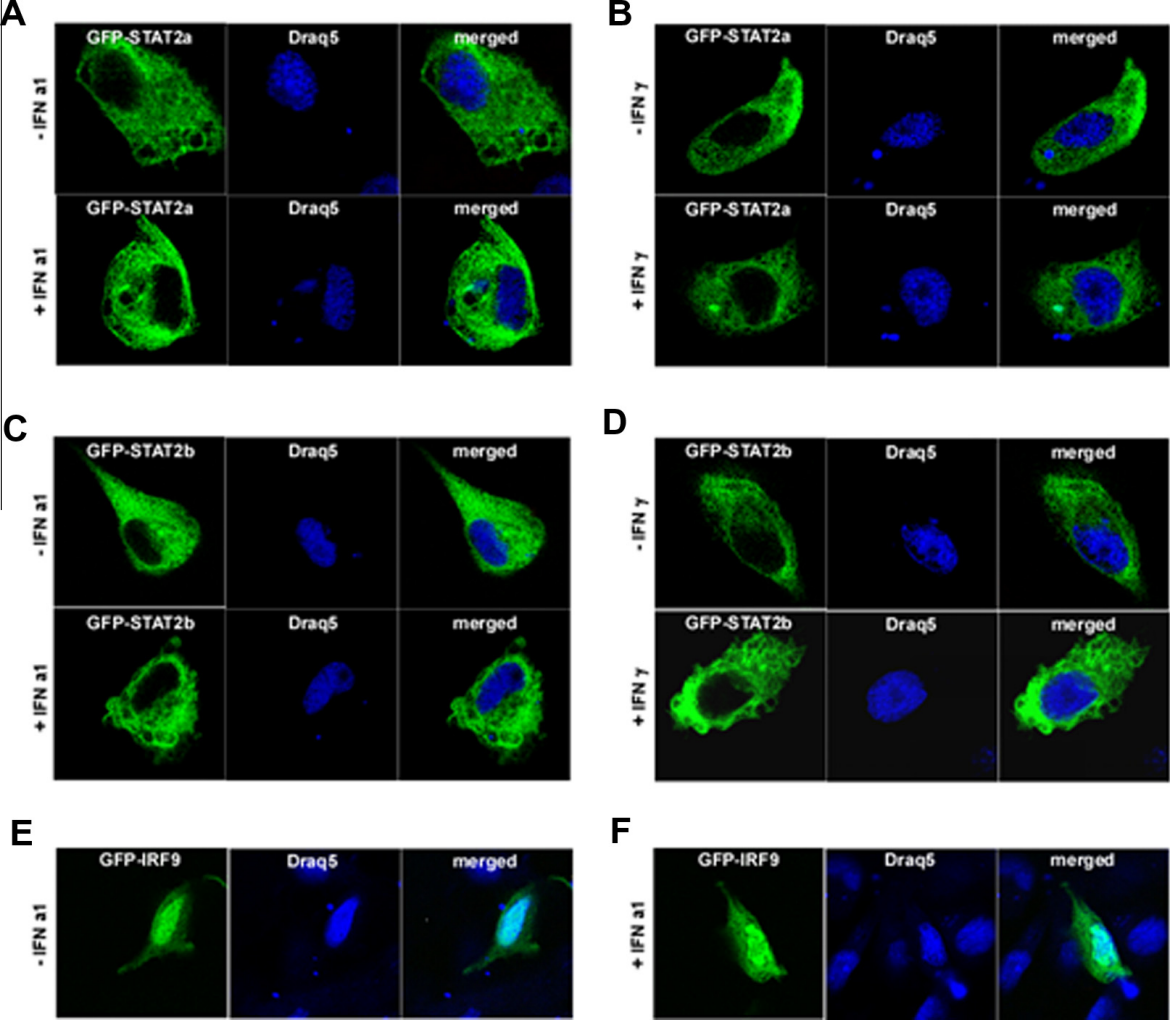
STAT2a and STAT2b localize in the cytoplasm, whereas IRF9 reside in the nucleus TO cells. (A) GFP-STAT2a non-stimulated (upper panel) and stimulated with IFNa1 (lower panel). (B) GFP-STAT2a non-stimulated (upper panel) and stimulated with IFNγ (lower panel). (C) GFP-STAT2b non-stimulated (upper panel) and stimulated with IFNa1 (lower panel). (D) GFP-STAT2b non-stimulated (upper panel) and stimulated with IFNγ (lower panel). (E) GFP-IRF9 non-stimulated and (F) stimulated with IFNa1. Cultured TO cells was transfected with the indicated constructs for 48 h. Subsequently, the cells were stimulated either with IFNa1 or IFNγ before fixation and counterstaining with Draq5 DNA dye to visualize nuclei and nucleic acids. Laser scanning confocal microscopy was done using Zeiss LSM 510 and high-magnification image analysis was done using a Zeiss LSM image browser.

**Fig. 8:**
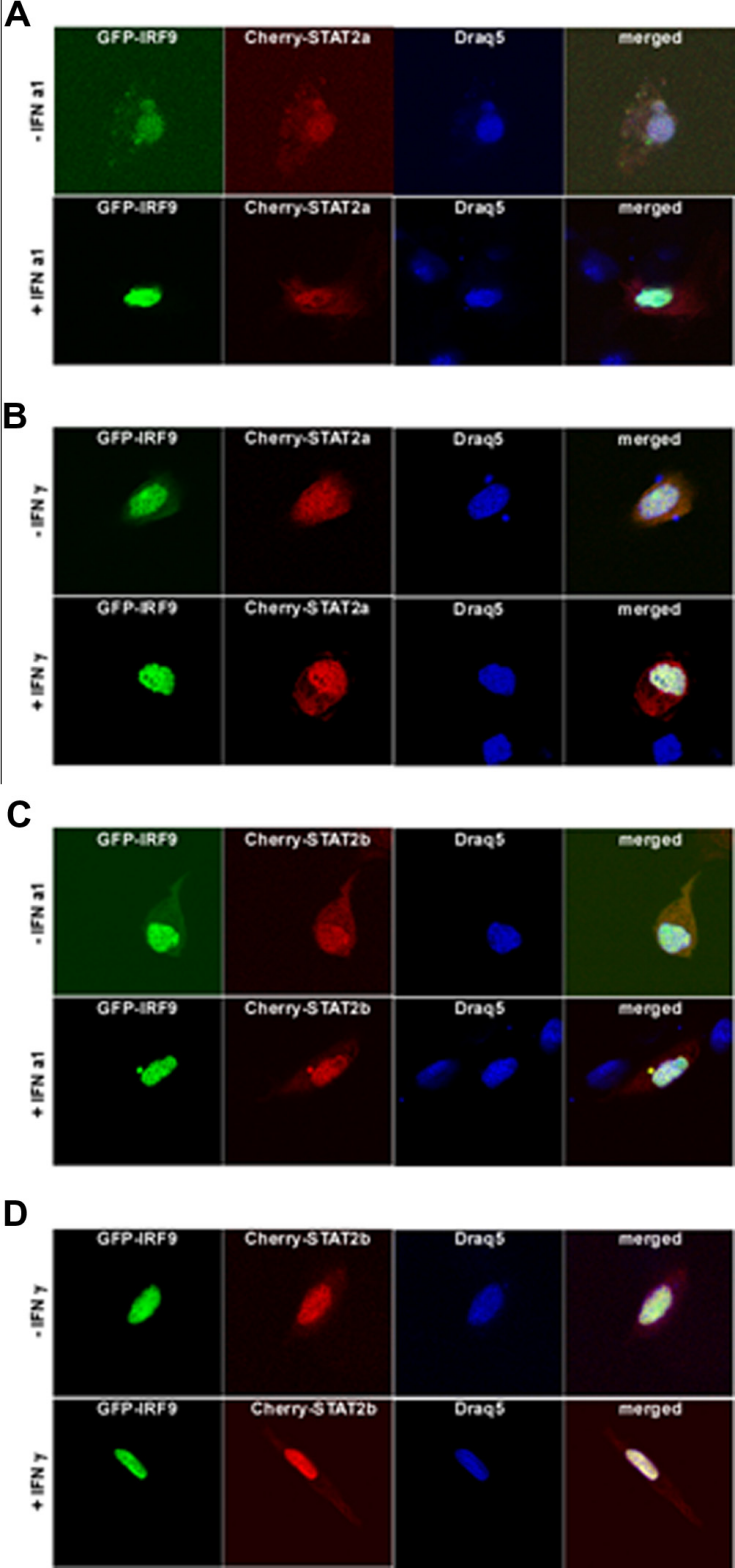
IRF9 alters the sub-cellular localization of STAT2a and STAT2b into nuclei of TO cells, independent of type I or type II IFN stimulation. (A) GFP-IRF9 and Cherry-STAT2a non-stimulated (upper panel) and stimulated with IFN a1 (lower panel). (B) GFP-IRF9 and Cherry-STAT2a non-stimulated (upper panel) and stimulated with IFNγ (lower panel). (C) GFP-IRF9 and Cherry-STAT2b non-stimulated (upper panel) and stimulated with IFN a1 (lower panel). (D) GFP-IRF9 and Cherry-STAT2b non-stimulated (upper panel) and stimulated with IFNγ (lower panel). Cultured TO cells were co-transfected with GFP-IRF9 together with Cherry-STAT2a or Cherry-STAT2b for forty-eight hours. Subsequently, the cells were either stimulated with IFNa1 or IFNγ before fixation and counterstaining with Draq5 DNA dye to visualize nuclei and nucleic acids. Laser scanning confocal microscopy was done using Zeiss LSM 510 and high-magnification image analysis was done using a Zeiss LSM image browser.

In order to study the sub-cellular localization, TO cells were transiently transfected with expression plasmids coding for GFP-tagged STAT2a, STAT2b, or IRF9 (Fig. 8A–F) and subjected to confocal microscopy. The results showed that both STAT2a and STAT2b resided in the cytoplasm of both stimulated and unstimulated cells (Fig. 7A–D). Even though IRF9 was mainly located in the nucleus, in some of the transfectants it was detectable in cytoplasm of both unstimulated and IFNa1 stimulated cells (Fig. 7E–F).

### 2.9 IRF9 direct STAT2a and STAT2b into the nucleus of TO cells

To explore the effect of IRF9 on sub-cellular localization of STAT2a or STAT2b, TO cells were co-transfected with an expression construct coding for IRF9 tagged with GFP together with expression constructs coding for either STAT2a or STAT2b tagged with the red fluorescent protein Cherry (Fig. 8A–D) and the transfected cells were stimulated either with IFNa1, or IFNγ or were left unstimulated. A control was included showing that also the Cherry tagged STAT2a and b, when expressed alone, was localized in cytoplasm in both unstimulated and IFN stimulated cells (Supplementary file 1). However, when co-transfected with IRF9, both STAT2a and STAT2b were detected in the nucleus independent of IFN stimulation (Fig. 8A–D), suggesting that IRF9 mediates nuclear import of STAT2. In some of the STAT2 and IRF9 co-transfected cells both proteins could be detected in the cytoplasm of unstimulated cells (Fig. 8). Shuttling between nucleus and cytoplasm has also been described in mammals [47].

### 2.10 STAT2a and STAT2b co-localize with STAT1a in the nucleus of IFN-stimulated TO cells

**Fig. 9:**
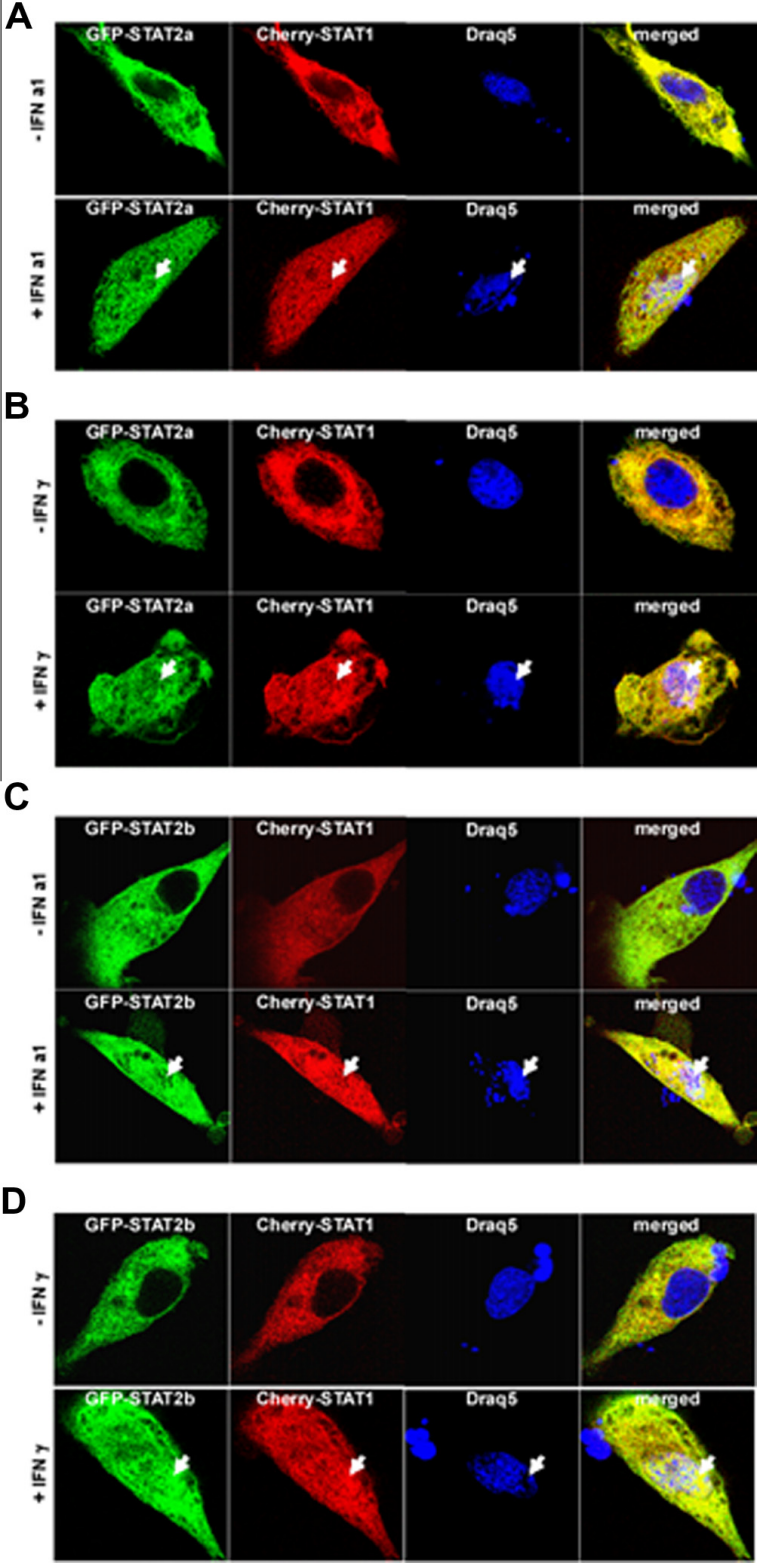
Sub-cellular localization of STAT2a and STAT2b together with STAT1a in salmon cells stimulated with type I or type II IFN. (A) GFP-STAT2a and Cherry-STAT1a non-stimulated (upper panel) and stimulated with IFNa1 (lower panel). (B) GFP-STAT2a and Cherry-STAT1a non-stimulated (upper panel) and stimulated with IFNγ (lower panel). (C) GFP-STAT2b and Cherry-STAT1a non-stimulated (upper panel) and stimulated with IFN a1 (lower panel). (D) GFP-STAT2b and Cherry-STAT1a non-stimulated (upper panel) and stimulated with IFNγ (lower panel). Cultured TO cells was co-transfected with the indicated constructs for forty-eight hours. Subsequently, the cells were either stimulated with IFNa1 or IFNγ before fixation and counterstaining with Draq5 DNA dye to visualize nuclei and nucleic acids. Laser scanning confocal microscopy was done using Zeiss LSM 510 and high-magnification image analysis was done using a Zeiss LSM image browser.

To study the effect of STAT1a expression on sub-cellular distribution of STAT2a and STAT2b, TO cells were co-transfected with an expression construct of STAT1a tagged with the red fluorescent protein Cherry together with expression constructs of STAT2a or STAT2b tagged with GFP (Fig. 9A–D). As expected STAT1a clearly co-localized with STAT2a in the cytoplasm of unstimulated cells, while upon IFN1a treatment STAT1a-STAT2a complexes were found in the nucleus (upper and lower panels in Fig. 9A) and a very similar pattern of distribution was found in cells treated with IFNγ (Fig. 9B). Like STAT2a, STAT2b did co-localize with STAT1a in the cytoplasm of unstimulated cells, and upon IFN stimulation (both IFNa1 and IFNγ) both proteins were found to accumulate in the nucleus as well as in cytoplasm (Fig. 9D).

### 2.11 Type I and type II IFNs show different profiles in their stimulatory effects on salmon STAT2a, STAT2b and IRF9 expression in TO cells

**Fig. 10:**
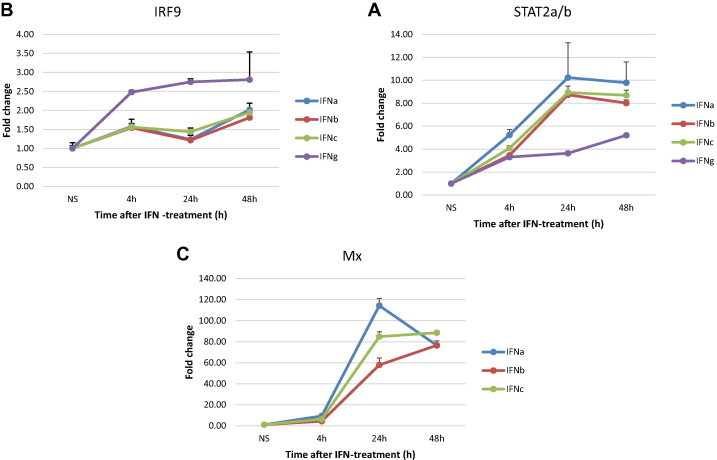
Expression kinetics of STAT2a, STAT2b and IRF9 in IFN-stimulated TO cells. TO cells were treated with 200 U/ml of IFNa1, IFNb and IFNc, and 100 ng/ml IFNγ for 4, 24 and 48 h and untreated cells were used as controls. The mRNA expression levels of STAT2 (A), IRF9 (B) and Mx (C) were measured by qPCR and transcripts levels were normalized against EF1αβ. The results are presented as fold increase relative to untreated cells in triplicate samples and are representative of two independent experiments.

To test the time-course expression of the STAT2a/b and IRF9 transcripts induced by IFNa1, b and c and by IFNγ treatment of TO-cells, a qPCR was performed. Additionally the expression of the interferon induced gene, Mx, was quantified to ensure that the stimulations had worked. The results showed that all the IFNs increased the expression of STAT2a/b until 24 h post stimulation. STAT2a/b was expressed at higher levels (10-fold induction) in IFNa1 treated cells compared IFNb and IFNc treated cells (about 8-fold induction) at this time-point (Fig. 10A). At 48 h post stimulation STAT2a/b expression was modestly reduced for all the type I IFNs. The kinetics of Mx expression was very similar to that of STAT2a/b for all three IFNs, but the fold induction was much higher (Fig. 10C), thus demonstrating that the IFN-stimulations had worked. For the IFNγ stimulated cells STAT2a/b were 4-fold induced at 24 h and continued to rise until 48 h (5-fold induction). IRF9 expression was modestly increased (1,5-fold) after 4 h in type I IFN treated cells, at 24 h the IRF9 levels were slightly down-regulated, and then their levels slightly increased (about 2-fold) to a maximum at 48 h (Fig. 10B). Upon IFNγ treatment elevated IRF9 mRNA levels were also detected after 4 h and increased further to a maximum at 48 h of stimulation (about 3-fold induction) (Fig. 10B).

### 2.12 IRF9 activates GAS element as result of IFNγ stimulation

Given effect of IFNγ on IRF9 and STAT2a/b expression in TO-cells combined with its ability to phosphorylate both STAT2a and STAT2b, it was interesting to study whether any of the factors studied here could mediate IFNγ responses. While some IFN-sensitive genes are activated by both type I and type II IFN, others, as also shown in fish [48], are preferably activated by type II IFN. In the case of genes sensitive to type II IFN, the transcription factor associates with a specific DNA sequences called GAS, [11]. GAS is comprised from a few nucleotides TTCN(2-4)GAA and constitutes interaction sequences for STAT1 dimers [49].

A commercial luciferase reporter from SABiosciences that contains tandem repeats of the GAS element was used for this study. Individual STAT1a, STAT2a, STAT2b, IRF9 constructs with a GFP-tag alone or in combinations were co-transfected with the GAS reporter plasmid in CHSE-214 cells. A GFP-vector without insert was used as a negative control. Transfectants were stimulated with IFNγ or left unstimulated and harvested 72 h post transfection (24 h post stimulation with IFNγ). Western blot of pooled lysates verified expression of all the transgenic proteins (results not shown).

**Fig. 11:**
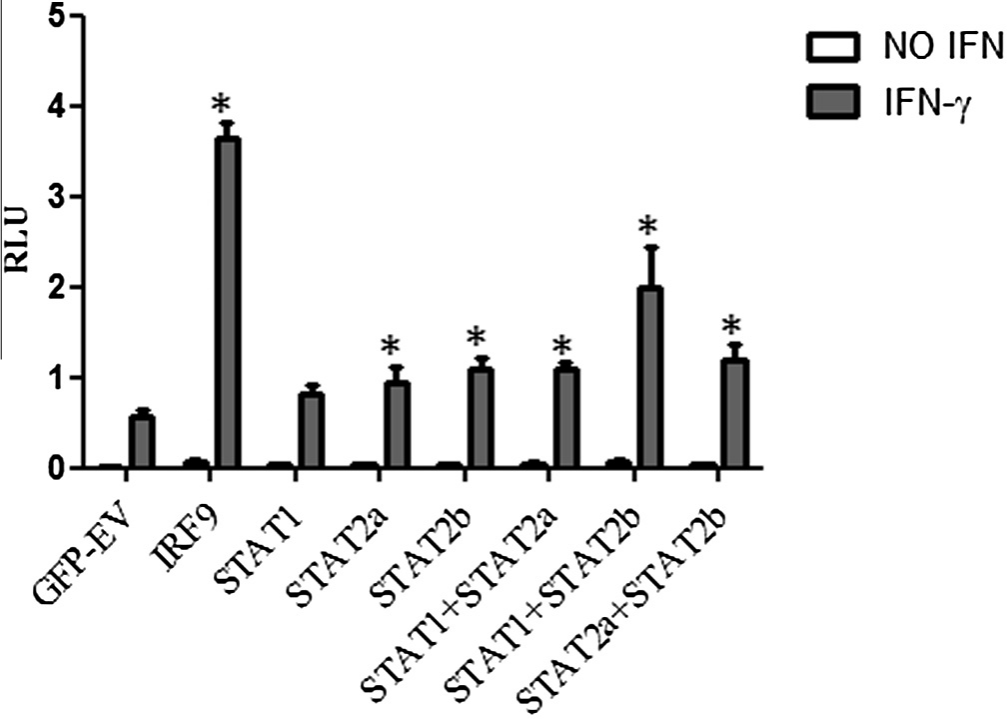
IRF9, STAT2a and STAT2b stimulate GAS promoter activity upon IFNγ stimulation. CHSE-214 cells transiently transfected with STAT1a, STAT2a, STAT2b, RF9 alone or in combination with each other and GFP-EV (empty vector) was used as control. Transfectants were left non-stimulated or were stimulated with 500 ng/ml of IFNγ 24 h prior to reporter gene assay analysis. Firefly luciferase activity was normalized with *Renilla* luciferase levels (*n* = 5) and the data are presented as relative luciferase units (RLU). The results are representative of two independent experiments. ^∗^ Represents values where difference compared to EV control is statistically significant.

The results presented in Fig. 11 show that addition of recombinant IFNγ induced a significant activation of the promoter compared to non-stimulated controls. Significant induction of luciferase activity was registered for overexpressed STAT2a, STAT2b and IRF9 alone, when compared to cells transfected with the plasmid control, and the most pronounced induction was detected for IRF9 transfected cells (*p* < 0.05). A modest induction of activity was also detected for cells transfected with STAT1a, although, the effect was not statistically significant when compared to the empty vector control. Of the combinations, STAT1a and STAT2b induced the highest GAS-promoter activity, which was significantly higher than each of the two alone, and the combination of STAT1a and STAT2a.

## 3 Discussion

Studies over the past 10 years have highlighted the important role IFNs play in providing protection against viral infections in bony fish. The antiviral responses are in large part achieved through the induction of genes by the JAK–STAT pathway, and homologs/orthologs to the *jak, stat* and *irf* genes have been described in many piscine species. However, the functional performance of the active proteins involved in the signaling pathways for type I and type II are less characterized in lower vertebrate species. In this paper we report cloning and characterization of two Atlantic salmon STAT2 isoforms, and one IRF9 homolog, as well as further characterization of a salmon STAT1a homolog [33]. The STAT2 sequences identified in this study had the same conserved structure as reported for other STAT molecules (Fig. 1A) and phylogenetic analysis showed that the salmon STAT2a and b clearly grouped with the other fish and vertebrate STAT2 genes with high bootstrap values. STAT2a and b had high aa identity with each other (>99%). A previously identified salmon STAT2 homolog (FJ173070) [35], has a TAD-domain almost identical to STAT2a, but has non-matching nucleotides in the coiled-coil domain, when compared to both STAT2a and STAT2b, thus representing another salmon STAT2 isoform. The STAT2b TAD domain is slightly shorter than the ones in STAT2a/c and its C-terminus sequence differs (Fig. 1). Additionally, for STAT2b cDNA its stop codon is proceed by a sequence identical to the C-terminus of STAT2a (results not shown). This could indicate that STAT2b is an alternative spliced variant of the STAT2a. Taken together, several distinct STAT2 orthologs exist in salmon, showing differences in their functional domains (i.e. TAD and coiled-coil), which also suggests that they possess distinct roles in JAK–STAT signaling. Profound divergences in the TAD domain are earlier reported between human and murine STAT2 [50]. The TAD domains from the two species were shown to bind both distinct and overlapping proteins in glutathione-S-transferase based affinity precipitation assays [50,51], suggesting that evolution has both conserved and altered the specific binding sites that are important for STAT2-dependent gene-regulation.

The salmon STAT2 and IRF9 genes showed ubiquitous tissue expression in healthy salmon. Furthermore, we show that in the salmon head kidney derived cell line TO the expression of these transcription factors is a subject to regulation by IFNs. An increase in STAT2a/b transcription was detected as early as 4 h of stimulation with IFNa1, b and c, as well as IFNγ, with the highest induction for type I IFNs at 24 h and for IFNγ at 48 h. This suggests that the STAT2s are under direct transcriptional regulation by IFNs. The IRF9 mRNA levels were slightly higher for IFNγ stimulated cells compared to cells treated by type I IFNs. It was not a surprise since it is already established that both GAS and GATE (IFNγ activated transcriptional element) motifs exist in all promoters of IRF9 from mammals and fishes, and in zebrafish, IRF9 is shown to be significant higher induced by zebrafish IFNγ2 than by zebrafish IFNφs [52].

The prevailing view in the literature, based on studies of mammalian species, is that the ISGF3 complex is essential for type I IFN signaling, and that STAT2 provides the essential TAD for this complex [53]. Interactions between STATs and JAKs, between STAT1 and STAT2 and between STATs and IRF9 are necessary for the transcription of type I IFN induced genes, and indeed these interactions have been detected in the mammalian models [9,54,55]. An important issue for this study was to compare the functional activity of the two salmon STAT2 molecules identified, and in particular their ability to interact with other members of the JAK–STAT signaling cascade, their nuclear trafficking and their ability to respond to different Atlantic salmon IFNs. Co-IP and confocal microscopy were used to study possible interaction between these factors in cell-lines, The co-IP results revealed evidence for interactions between the two STAT2s and STAT1a, ssTyk2-1 and IRF9 (Fig. 6). As transcription factors the STATs must gain access to the nucleus, and it was therefore of interest to study their cellular trafficking when overexpressed alone or in combinations. Both the salmon STAT2 molecules remained in the cytoplasm in untreated and IFN-stimulated TO cells. However, when co-expressed with IRF9 both molecules accumulated in the nucleus and colocalized with IRF9, also in the absence of IFN-stimulation. This observation provide further evidence for the existence of an IRF9–STAT2 complexes, and agrees with earlier reported findings in mammals where unphosphorylated STAT2 shuttles between the nucleus and cytoplasm [56]. Studies of human STAT2 have shown that nuclear translocation of unphosphorylated STAT2 is dependent on its association with IRF9 [47]. IRF9 directly interacts with the coiled-coil domain of STAT2, and the NLS-signal in the N-terminus of IRF9 directly interacts with importins, which are proteins that mediates movement through the nuclear pore complex. An aa sequence stretch which was enriched in basic aa (L, R) was found in the salmon IRF9 sequence and most probably this represents an intrinsic NLS responsible for the shuttling of IRF9–STAT2 complex to the nucleus.

It has been previously shown that STAT1a is tyrosine phosphorylated as result of IFNa1 and IFNγ stimulation – a precondition for dimerization and translocation [33]. We here pinpoint the salmon STAT1a phosphorylation site to SH2 domain of the molecule. While wild-type STAT1a was phosphorylated by IFNa1, IFNc and IFNγ, a point mutated construct (STAT1aY695A) was not. Based on this, Y695, corresponding to Y791 in human (GenBank accession no. NM_007315), mouse (GenBank accession no. U06924) and rat (GenBank accession no. AF205604) STAT1, was identified as the salmon STAT1a tyrosine phosphorylation site. One interesting observation was the fact that stimulation with neither of the IFNs lead to phosphorylation of an ectopically expressed STAT1a deletion construct which possessed the SH2 domain, but lacked the other STAT1a domains. This was surprising since Y695 is situated in the SH2 domain. One possible explanation could be that the SH2 domain folding might be affected when expressed alone and/or other domains might also be important in facilitating a functional interaction between STAT1a and JAK molecules or between STAT1a and IFN-receptors.

Both STAT2a and STAT2b were tyrosine phosphorylated upon stimulation with IFNa1 and less expected, also by IFNγ (Fig. 4). The phosphorylation was not as pronounced as for STAT1a, but it was still higher than that of the control. Confocal microscopy results showed that STAT2a and STAT2b did translocate to the nucleus when co-transfected with STAT1a and stimulated with type I IFN (Fig. 9). This was expected since STAT2 is known to be involved in type I IFN signaling [53] and correlates with findings from turbot, *Scophthalmus maximus* where STAT2 nuclear localization was detected in poly I:C treated TK cells [57]. In IFNγ stimulated cells, using the same set-up, fractions of STAT2a and STAT2b translocated to the nucleus when co-expressed with STAT1a (Fig. 9). This, together with the data demonstrating phosphorylation of the STAT2 isoforms upon IFNγ stimulation suggests that STAT2 has a role in Atlantic salmon type II IFN signaling. In order to study the functional significance of STAT2 phosphorylation and nuclear localization upon IFNγ stimulation, a GAS firefly reporter gene construct was used that contains tandem repeats of a GAS-element. Ectopically expressed STAT2a, STAT2b and IRF9 caused significant upregulation of luciferase activity in IFNγ stimulated cells compared to empty vector control and, of notice, the highest response was detected in cells transfected with IRF9 alone. STAT1a alone was not able to modulate neither basal luciferase activity nor IFNγ stimulated activity. Furthermore, STAT2b co-transfected with STAT1a caused significantly higher reporter activity than STAT2a co-expressed with STAT1a. IRF9 in combinations with any of the STAT constructs did not further enhance reporter gene activity compared to IRF9 alone (results not shown). The results presented here suggest that salmon STAT2s, and in particular the STAT2b isoform, and also IRF9, are involved in IFNγ signaling through the GAS-element. The results may indicate that overexpression of STAT2 or IRF9 combined with IFNγ-stimulation may induce formation of specific complexes that can bind to the GAS-elements and serve as transcription factors. The highest divergence between STAT2a and STAT2b was found in the TAD-domain, and also most of the divergence between mammalian and fish STAT2 lies in this domain. The STAT2-TAD has been shown to bind and recruit transcriptional co-activators [50,58–60], and it is thus possible that salmon STAT2 in a STAT1:STAT2 heterodimer recruit activators important for GAS activation. The presented data may suggest that the salmon STAT2 TAD and also IRF9 play roles in IFNγ induced signaling in a manner which makes type II IFN signaling in bony fish distinct from the mammalian pathway. In addition to the canonical STAT1 homodimer signaling pathway there is evidence of alternative STAT1 independent complexes/pathways which trigger different transcriptional programs [61]. These studies including the one reported here show that the JAK/STAT signaling are much more diverse than originally thought.

## 4 Experimental procedures

### 4.1 Fish

For organ sampling healthy Atlantic salmon (*S.* *salar*) strain Aquagen standard (Aquagen, Kyrksæterøra, Norway), were obtained from the Tromsø Aquaculture Research Station (Tromsø, Norway). The fish with a mean weight of approximately 117 g were kept in tanks supplied sea water at 5 °C, 24 h light and were fed on commercial, dry food (Skretting, Stavanger, Norway). All experiments were approved by the national committee for animal experimentation (Forsøksdyrutvalget, Norway) and performed according to its guidelines. The fish were anesthetized by Benzoac 20% (0.6 ml/l) to eutanasi.

### 4.2 Cloning of STAT2 and IRF9 and plasmid construction

Primers for PCR amplification (Table 1) were constructed based on the full sequences of Atlantic salmon STAT2 (GenBank accession no. FJ173070) [35] and Atlantic salmon IRF9 (NM 001173719) [36] already registered in the GeneBank. Mixed cDNA derived from healthy Atlantic salmon ovaries and head kidney was used as template for the PCR reactions (Hansen and Jørgensen, 2007). The PCR products were inserted into the Gateway compatible pENTR/D-TOPO vector (Invitrogen) and sequenced using the BigDye chemistry and a 3100 Gene Analyzer (Applied Biosciences). The inserts were then transferred to eukaryotic expression vectors (pDest-FLAG, pDest-EGFP and pDest-mCherry) by Gateway recombination using LR clonase II enzyme mix (Invitrogen) following the manufacturer’s instructions and plasmids used are listed in Table 2. Four truncated derivatives of STAT1 (GQ325309) were made using primers listed in Table 1 and sequences were verified and transferred to expression vectors using the same procedure as described above. A point mutant was made by using the QuickChange site-directed mutagenesis kit (Stratagene) according to the manufacturer‘s instructions and primers designed based on STAT1a sequence (Table 1).

### 4.3 Sequence and phylogenetic analysis

Relevant sequences for alignment and phylogenetic analysis of STAT and IRF family members were collected. Sequences alignments were performed using the BioEdit program (http://www.mbio.ncsu.edu/bioedit/bioedit.html). The deduced open reading frames (ORFs) of the STAT2a, STAT2b and IRF9 were analyzed using the NCBI conserved domains database [37] and ClustalW alignment. A phylogenetic tree for STAT2 was constructed using MEGA 5.1 program (http://www.megasoftware.net/) and built using the neighbor-joining algorithm. Support for internal branching was assessed by bootstrap repetitions and bootstrap values were set to 500.

### 4.4 Cell cultures

HEK293T cells were cultured in Eagle minimal essential medium (EMEM) containing GlutaMAX (Invitrogen) supplemented with 100 μg/ml streptomycin, 60 μg/ml penicillin, 1% non-essential amino acids and 10% FBS at 37 °C and 5% CO_2_. Chinook salmon embryo cells (CHSE-214) [38] were cultured in EMEM containing GlutaMAx supplemented with 100 μg/ml streptomycin, 60 μg/ml penicillin, 1% non-essential amino acids and 8% FBS at 20 °C and 5% CO_2_. Atlantic salmon TO cells [39] cells were cultured in EMEM containing GlutaMAx supplemented with 100 μg/ml streptomycin, 60 μg/ml penicillin, 1% non-essential amino acids and 5% FBS at 20 °C and 5% CO_2._

### 4.5 Cell stimulations

Both for phosphorylation and for real-time quantitative PCR (qPCR) studies, TO and CHSE-214 cell lines were stimulated with 500 ng/ml IFNγ or 200 U/ml of IFNa1, IFNb and IFNc at indicated times. Recombinant Atlantic salmon IFNa1, IFNb, and IFNc were produced in HEK293 cells and the titers were estimated as described elsewhere [40,41]. Recombinant IFNγ was expressed as described by Zou et al. [42]. All dilutions for stimulations were made in EMEM supplemented with FBS, antibiotics and nonessential amino acids as described above. Control samples consisted of cell lines incubated with culture medium alone.

### 4.6 Antibodies

The antibodies used in this study included a monoclonal FLAG® M2 antibody from Sigma Aldrich, a polyclonal anti-GFP antibody from Abcam, a monoclonal anti-phospho-tyrosine 4G10 antibody from Millipore, goat-anti-rabbit IgG-HRP and goat-anti-mouse IgG-HRP antibodies from Santa Cruz Biotechnology, goat-anti-Rabbit IRDye 800 CW and goat-anti-Mouse IRDye 680 RD antibodies from LI-COR. All antibodies were diluted in blocking buffer at concentrations recommended by manufacturer.

### 4.7 Immunoprecipitations

Co-immunoprecipitation (co-IP) was used to study possible interactions between the different STATs, the STATs and IRF9 and the STATs and Tyk2. In these experiments, different combinations of GFP- and FLAG-tagged STAT1a, STAT2a, STAT2b, IRF9 and ssTyk2-1 were used. For co-transfection, HEK-293T cells were seeded into 6-well plates (1 × 10^6^ cells/well) and transfected with 3 μg plasmid DNA (1.5 μg of each plasmid) using Lipofectamine 2000 Transfection Reagent (Life technologies) and harvested 48 h post transfection using HA-buffer (50 mM Tris–HCl, pH 7.5, 150 mM NaCl, 2 mM EDTA, 1 mM EGTA, 1% [v/v] Triton X-100) added protease inhibitor (Complete EDTA-free; Roche Applied Science). Cell lysates were cleared with centrifugation (15,000*g* in 15 min) and incubated with anti-FLAG M2 affinity gel (Sigma–Aldrich) (1 h, 4 °C). The bound proteins with the interacting partners were eluted with 2× LDS sample buffer (Invitrogen) and were subjected to SDS–PAGE and visualized by Western blotting (see below).

Immunoprecipitation (IP) analysis was used to study changes in tyrosine phosphorylation upon stimulation. CHSE-214 cells were seeded into 6-well plates (5 × 10^5^ cells/well), transfected with 3 μg GFP-tagged plasmids using TransIT-LT-1 (Mirus) and stimulated with IFNs for 30 min before they were harvested 72 h post transfection in lysis buffer (20 mM Tris–acetate pH 7.0, 0.27 M sucrose, 1 mM EDTA, 1 mM EGTA, 1 mM sodium orthovanadate, 10 mM β-glycerophosphate, 50 mM sodium fluoride, 5 mM sodium pyrophosphate, 1% [v/v] Triton X-100, 0.1% [v/v] 2-mercaptoethanol) added protease inhibitor (Complete EDTA-free; Roche Applied Science). Cell lysates were cleared as described above and incubated with anti-GFP at 4 °C for 1 h and antibody–antigen complexes were precipitated with pre-blocked protein A-agarose beads (Millipore) and eluted with 2× LDS sample buffer (Invitrogen). The eluted proteins were subjected to SDS–PAGE and visualized by Western blotting (see below).

### 4.8 SDS–PAGE and Western blotting

The eluates from the IPs/co-IPs were directly subjected to SDS–PAGE using NuPAGE Novex Bis-Tris 4–12% gels (Life Technologies) and then proteins were transferred to PVDF membrane and blocked for one h in 5% fat free dry milk or 5% BSA in TBS-T and then incubated with the primary antibody at 4 °C, then the membranes were treated with the secondary antibody for 1 h.

In this study two different imaging systems were used; the first one was KODAK Image Station 4000MM Digital Imaging System where membranes were blocked with dry milk or BSA, secondary antibodies were HRP conjugated and the blots were developed using SuperSignal West Pico Chemiluminesccent Substrate (Pierce). The second system was Odyssey CLx infrared imaging system where specific blocking buffer from LI-COR was used and the secondary antibodies were infrared dye conjugated. The membranes were stripped by incubating in 0.2 M NaOH in 15 min and process was repeated for the primary antibody specific for the precipitated factor.

### 4.9 Immunofluorescence microscopy

To examine subcellular localization of expressed constructs in fish cells, Atlantic salmon TO cells were seeded with a density of 7 × 10^4^ cells per well in 8-well Nunc culture dishes (Thermo Scientific). The cells were transiently transfected with constructs tagged with the GFP, alone or in combination with constructs tagged with the monomeric red fluorescent protein Cherry (Table 2), using the transfection reagent TransIT-LT1 (Mirus) as described by the manufacturer.

Forty-eight hours post transfection the cells were stimulated with recombinant IFNa1 (500 U/μl) or IFNγ (300 ng/μl) for 45 min. The cells were briefly rinsed once with phosphate buffered saline (PBS) before fixation with 4% (v/v) methanol-free formaldehyde (Thermo Scientific) for 20 min on ice, and subsequently washed twice with PBS. For permeabilization of cellular membranes, the cells were added 0.5% of the detergent Triton X-100 for 10 min on ice. To visualize nuclei and nucleic acids, the cells were counterstained with DRAQ5 far-red fluorescent cell permeable DNA dye (Cell Signaling Technologies) for 5 min on ice and subsequently washed twice with PBS. Laser scanning microscopy was done using a Zeiss LSM 510 META confocal microscope version 3.2 SP2 (Carl Zeiss GmbH) and high-magnification confocal image analysis was done using a Zeiss LSM image browser version 4.2.0.121 (Carl Zeiss MicroImaging GmbH).

### 4.10 Total RNA extraction, cDNA synthesis and real time PCR analysis

Total RNA was extracted from Atlantic salmon tissues (heart, gill, intestine, spleen, posterior kidney, head kidney, liver, skin, thymus and muscle) and from TO cells using the RNeasy Mini Kit (Qiagen, Hilden, Germany) in combination with TRIzol® (Invitrogen).The RNA yield was determined using Nanodrop ND-1000 Spectrophotometer (Nanodrop Tec., Wilmington, DA, USA). For each sample, 200 ng of the total RNA was used for cDNA synthesis using random hexamers in a 10 μl reaction volume applying the TaqMan Reverse Transcription Reagents kit as recommended by the manufacturer’s (Applied Biosystems). The synthesized cDNA was then diluted 10X, using RNase-free water and used as template for qPCR. The qPCR was performed in duplicates and each reaction mixture contained 2.5 μl of a diluted cDNA template (5 ng of reverse transcribed RNA), 10 μl of Fast SYBR Green Master Mix (2×; Applied Biosystems), and 1 μl primer mix equivalent to 150 nM forward and reverse primers (Table 2) in a 20 μl reaction volume. The amplification profile consisted of an initial denaturation step at 95 °C for 20 s, followed by 40 cycles of 95 °C for 3 s and 60 °C for 60 s followed by melting curve from 60 °C to 95 °C in a 7500 Fast Real-Time PCR System (Applied Biosystems). Amplification specificity was analyzed using dissociation curves and the relative expression values were normalized against the levels of Atlantic salmon EF1A_B_ and calculated using the method described by Pfaffl [43]. For tissue samples the expression levels for each tissue are presented as change in expression relative to EF1A_B_ as described previously [34].

### 4.11 Reporter gene assay

CHSE cells grown in 96-well plates were co-transfected with plasmids containing the GAS reporter (SA Biosciences), the pRL-SV40 Vector (Promega) and constructs coding for GFP-tagged IRF9, STAT2a, STAT2b or an empty vector containing only GFP (25 ng of each plasmid/well) using TransIT-LT-1 (Mirus) transfection reagents according to the manufacturer’s protocol. The cells were incubated for 48 h before being stimulated with IFNγ (500 ng/ml), or left unstimulated for another 24 h. After the stimulation period, the cells were washed with PBS and lysed in lysis buffer (20 μl/well) for the luciferase luminescence assay using the Dual Luciferase Reporter Assay System (Promega), according to the manufacturer’s protocol. The firefly and *Renilla* luciferase activity was measured in a Luminoscan RT luminometer (Labsystems OY). All samples for the luciferase assay were set up in five replicates. The *Renilla* luciferase levels were used to normalize firefly luciferase values, and results were presented as relative luciferase induction units (RLU). The remaining 10 μl of the lysates was dissolved in 10 μl of 2× NuPAGE LDS loading buffer with reducing agent (Invitrogen). The replicates were pooled and the samples were subjected to SDS–PAGE and visualized by Western blotting to control the expression of mentioned proteins (result not shown).

### 4.12 Statistics

Statistical analysis was done using one way ANOVA followed by Turkey’s multiple comparison test for differences between treatments using the GraphPad prism software. Data with *p* values less that 0.05 (*p* < 0.05) is reported as statistically significant.

## Figures and Tables

**Supplementary Fig. S1:**
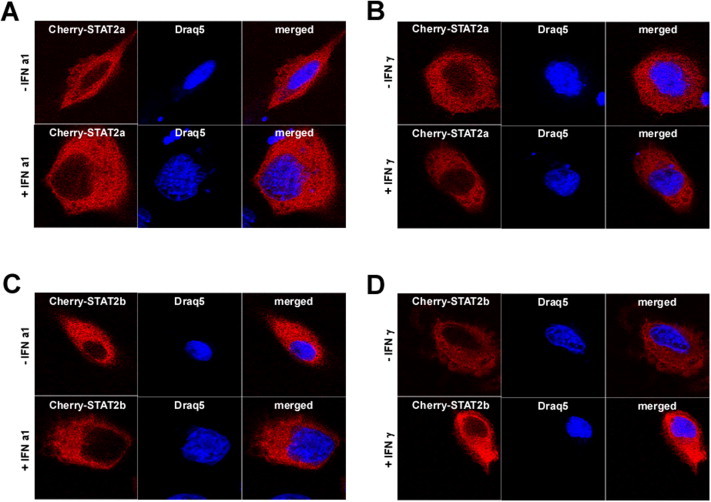


**Table 1:** Primers used for cloning and qPCR.

Primer name	Application	Sequence	Accession No.
Point mut STAT1aY695A fw	Cloning	CAACATGATGCCCATGGCGCCAGACGTGTTCG	GQ325309
Point mut STAT1aY695A R	Cloning	CGAACACGTCTGGCGCCATGGGCATCATGTTG	
STAT1a Δ125–758 fw	Cloning	CACCATGGCCCAGTGGTGCCAGCTGCAG	
STAT1a Δ125–758 R	Cloning	TCAGTCGGTCTCTATGCTTTTGGCAGCA	
STAT1a Δ1–320 Δ563–758 fw	Cloning	CACCATGCCCTGTATGCCCACACATCC	
STAT1a Δ1–320 Δ563–758 R	Cloning	TCAGATGTCTCTTGATGAGGTCC	
STAT1a Δ1–574 Δ733–758 fw	Cloning	CACCATGCTGTGGATCGAAGCTATCCT	
STAT1a Δ1–574 Δ733–758 R	Cloning	TCAAGGGGTGAACTTCTGACACTG	
STAT1a Δ1–574 fw	Cloning	CACCATGCTGTGGATCGAAGCTATCCT	
STAT1a Δ1–574 R	Cloning	TCAGTTGCGGTCCAAGTCAGGTGT	
STAT2 fw	Cloning	CACCATGGCCCAGTGGGAGAAGCT	FJ173070
STAT2 R	Cloning	CTACTAATCTTCTCCAGCTCTTCGGAG	
IRF9 fw	Cloning	CACCATGGCATCTGGGAGAGTTCGCTCAAC	NM_001173719
IRF9 R	Cloning	TCAGGGGTAAGGCACTGGTACC	NM_001173719
EF1A_B_ fw	qPCR	TGCCCCTCCAGGATGTCTAC	AF321836
EF1A_B_ R	qPCR	CACGGCCCACAGGTACTG	
Mx fw	qPCR	GATGCTGCACCTCAAGTCCTATTA	U66475
Mx R	qPCR	CGGATCACCATGGGAATCTGA	
STAT2a/b fw	qPCR	GAGGAACAACAAGATGAGTTTGATTTTAA	
STAT2a/b R	qPCR	CTCATACCGGACAACATGCTC	
IRF9 fw	qPCR	TCTGAAAGCAGTGGGTCAGGATGT	NM_001173719
IRF9 R	qPCR	ACGTTCTCAGTCCAGAGTGT	NM_001173719

**Table 2:** Plasmids used in this study.

Name	Vector	Application	Accession No.
STAT1a	pDEST-GFP/pDEST-FLAG/pDEST-Cherry	IP, co-IP, RGA, confocal microscopy	GQ325309
STAT1aY695A	pDEST-GFP/pDEST-FLAG	IP, co-IP, RGA	
STAT1a Δ125–758	pDEST-GFP/pDEST-FLAG	IP, co-IP	
STAT1a Δ1–320 Δ563–758	pDEST-GFP/pDEST-FLAG	IP, co-IP	
STAT1a Δ1–574 Δ733–758	pDEST-GFP/pDEST-FLAG	IP, co-IP	
STAT1a Δ1–574	pDEST-GFP/pDEST-FLAG	IP, co-IP	
STAT2a	pDEST-GFP/pDEST-FLAG/pDEST-Cherry	IP, co-IP, RGA, confocal microscopy	KJ155789
STAT2b	pDEST-GFP/pDEST-FLAG/pDEST-Cherry	IP, co-IP, RGA, confocal microscopy	KJ155790
IRF9	pDEST-GFP/pDEST-FLAG	IP, co-IP, RGA	NP_001167190
ssTyk2-1	pDEST-GFP/pDEST-FLAG	co-IP	KF021282

**Table 3:** Percent aa-identity between the different Atlantic salmon STAT2 homologs and STAT2 from other organisms.

Organism	Accession No.	Atlantic salmon STAT2a (%)	Atlantic salmon STAT2b (%)
Atlantic salmon STAT2a	KJ155789	–	96.27
Atlantic salmon STAT2b	KJ155790	96.27	–
Atlantic salmon STAT2c	FJ173070	92.39	88.03
Goldfish STAT2	JQ804927	59.9	61.12
Zebrafish STAT2	NM_001271801	57.69	58.76
	XM_688485		
Western clawed frog STAT2	NM_001129930	43.4	43.53
Human STAT2-2	NM_198332	40.34	41.12
Human STAT2-1	NM_005419	40.34	41.12
Cow STAT2	NM_001205689	40.47	40.87
	XM_588270		
Wild boar STAT2	AB004061	40.1	40.75
Rat STAT2	NM_001011905	38.75	39.63
	XM_222301		
Mouse STAT2	NM_019963	38.75	38.26
	XM_990913		
